# Trap-TRAP, a Versatile Tool for Tissue-Specific Translatomics in Zebrafish

**DOI:** 10.3389/fcell.2021.817191

**Published:** 2022-01-31

**Authors:** Jorge Corbacho, Estefanía Sanabria-Reinoso, Lorena Buono, Ana Fernández-Miñan, Juan R. Martínez-Morales

**Affiliations:** Centro Andaluz de Biología del Desarrollo- CABD (CSIC/UPO/JA), Seville, Spain

**Keywords:** enhancer-trap, Gal4/UAS, zebrafish, TRAP, translatome

## Abstract

Developmental and physiological processes depend on the transcriptional and translational activity of heterogeneous cell populations. A main challenge in gene expression studies is dealing with this intrinsic complexity while keeping sequencing efficiency. Translating ribosome affinity purification (TRAP) methods have allowed cell-specific recovery of polyribosome-associated RNAs by genetic tagging of ribosomes in selected cell populations. Here we combined the TRAP approach with adapted enhancer trap methods (trap-TRAP) to systematically generate zebrafish transgenic lines suitable for tissue-specific translatome interrogation. Through the random integration of a GFP-tagged version of the large subunit ribosomal protein L10a (EGFP-Rpl10a), we have generated stable lines driving expression in a variety of tissues, including the retina, skeletal muscle, lateral line primordia, rhombomeres, or jaws. To increase the range of applications, a *UAS:TRAP* transgenic line compatible with available Gal4 lines was also generated and tested. The resulting collection of lines and applications constitutes a resource for the zebrafish community in developmental genetics, organ physiology and disease modelling.

## Introduction

The precise combination of repressed and activated genes determines the identity and transcriptional state of the cells, and thus controls their shape, mechanical properties, physiology, pathology, and survival. Genome-wide analysis of the transcriptome in different tissues provides very helpful information on the cells’ state through time. The emergence of Next Generation Sequencing (NGS) technologies allowed generating large volumes of sequencing data per run, offering whole-genome coverage at reduced costs. Among the numerous NGS applications, RNA-seq has become a method of choice in transcriptomics due to its high reproducibility, unbiased detection, single nucleotide resolution and quantitative estimation over a large dynamic range of gene expression (Wang et al., 2009). Despite its numerous advantages, RNA-seq analytical power is significantly diminished when complex tissues, such as the brain, are examined. Using an “en masse” approach, gene expression profiles cannot be assigned to any specific cell type, but reflect averaged gene expression across the entire tissue. Many of these disadvantages have been overcome by single-cell RNA sequencing (scRNA-seq) technologies, which permit the characterization of heterogeneous cell populations, making possible to detect the signature of rare cell types or transient cellular states (Chen et al., 2019). However, due to the low amount of starting material, scRNA-seq methods have their own technical limitations such as loss of spatial information, low capture efficiency, and frequent dropout events (Kulkarni et al., 2019). In consequence, only a fraction of the transcriptome of each cell can be detected by scRNA-seq, and the technical noise is higher than in bulk RNA-seq (Kolodziejczyk et al., 2015). Therefore, the in-depth transcriptomic characterization of a given cell type still depends on our ability to isolate them either through micro-dissection or using fluorescence-activated cell sorting (FACS) protocols.

Unfortunately, severe dissociation procedures such as those required before FACS, may distort gene expression inducing a cellular response to stress (Richardson et al., 2015; O'Flanagan et al., 2019). As an alternative to flow cytometry, a number of approaches have been developed to directly label RNA, such as TU-tagging (Miller et al., 2009), or RNA-binding proteins (Roy et al., 2002; Heiman et al., 2008). Among them, the translating ribosome affinity purification (TRAP) technology stands out due to its low toxicity and its suitability in both, vertebrates and *Drosophila* (Doyle et al., 2008; Heiman et al., 2008; Thomas et al., 2012; Bertin et al., 2015). This method is based on cell-type specific ribosome tagging, by expressing a GFP-tagged version of the large subunit ribosomal protein L10a (EGFP-Rpl10a) under the control of a tissue-specific promoter of choice. Then, labelled polyribosomes can be affinity purified to specifically pull-down associated mRNAs, which are a precise representation of translated genes in the cell population of interest. The TRAP methodology provides important advantages, as it does not require tissue fixation or dissociation and the cells of interest are marked, which facilitates in parallel imaging studies. Moreover, analysing the translating mRNA pool provides a closer representation of the protein content than the examination of the whole mRNA profile (Heiman et al., 2008; Heiman et al., 2014).

In zebrafish, different laboratories have successfully implemented the TRAP approach using tissue-specific drivers. They include the promoters of the genes *tyrp1* for melanocytes (Tryon et al., 2013), *cmlc2* for cardiomyocytes (Fang et al., 2013), *actc1b* for skeletal myocytes (Housley et al., 2014), or *lyz* for neutrophils (Houseright et al., 2020). One of the main disadvantages of the TRAP methodology is precisely the need to create a customized transgenic line for each cell type to be analysed. This is a laborious and technically challenging procedure that may discourage many zebrafish laboratories; particularly when no suitable drivers are available for the population of interest.

To overcome some of these limitations, we have developed a strategy combining the TRAP technology with a traditional enhancer trap approach: here referred as *trap-TRAP*. Enhancer trap methodologies allow the random genomic insertion of a reporter gene to capture the activity of nearby cis-regulatory elements (O'Kane and Gehring, 1987). In zebrafish, the adaptation of transposons, in particular that of the medaka element Tol2, has greatly improved the efficiency of enhancer trap approaches, allowing the generation of collections of stable enhancer trap lines (Kawakami et al., 2000; Kawakami, 2005; Korzh, 2007). Using our *trap-TRAP* approach, which takes advantage of the Tol2 system to generate random insertions of an *eGFP-rpl10a* cassette, we have isolated 33 tissue specific lines in a pilot screen. Furthermore, by placing the eGFP-rpl10a fusion under the control of the UAS element, we have also combined the TRAP technique with the Gal4/UAS transcriptional activation system (Scheer and Campos-Ortega, 1999). This allows expanding the applicability of the TRAP method to the entire collection of Gal4 drivers available in zebrafish (Scott et al., 2007; Asakawa and Kawakami, 2009). Taken together, our approaches allow the systematic generation of transgenic lines for efficient tissue-specific translatome interrogation in zebrafish.

## Materials and Methods

### Fish Maintenance

The zebrafish (*Danio rerio*) AB/Tübingen (AB/TU) wild-type strain, and the transgenic line *Tg[Rx3:Gal4:UAS:RFP]* (Weiss et al., 2012) were maintained under standard conditions at 28°C. Embryos and larvae were kept in E3 medium (5 mM NaCl, 0.17 mM KCl, 0.33 mM CaCl2, 0.33 mM MgSO4) supplemented with Methylene Blue (Sigma) at 28°C and staged according to somite number and morphology (Kimmel et al., 1995). Animal experiments were carried out according to ethical regulations. Experimental protocols have been approved by the Animal Experimentation Ethics Committees at the Pablo de Olavide University and CSIC (license number 02/04/2018/041).

### TRAP Vectors Construction

To develop the different approaches carried out in this work, two different constructs were built, based on the *Tol2* transposition system (Kawakami et al., 2004): *Tol2_trap:TRAP* and *Tol2_UAS:TRAP*. Both constructs were obtained through modifications of the Tol2-zTRAP plasmid, which contains the eGFP-rpl10a fusion gene flanked by Tol2 sites (Tryon et al., 2013). The *Tol2_trap:TRAP* construct was generated by inserting the *gata2p* minimal promoter from the ZED vector (Bessa et al., 2009) into the *Tol2-zTRAP* multiple cloning site using *SalI* and *BamHI* restriction enzymes. The *Tol2_UAS:TRAP* plasmid was built by inserting the UAS fragment from the Tol2kit p5E_UAS plasmid (Kwan et al., 2007) into the *Tol2-zTRAP* multiple cloning site using *BamHI* and *NheI*. To obtain the *Tol2_Vsx2.2:TRAP* plasmid, a 5 Kb region of the *vsx2.2* promoter (Nicolas-Perez et al., 2016) was also introduced in the *Tol2-zTRAP* multiple cloning site using *NheI*.

### Translating Ribosome Affinity Purification, Sequencing and Data Analysis

The polyribosome-attached RNA isolation was performed as previously described (Heiman et al., 2008) with some modifications. As starting material, *300 Tg(vsx2:TRAP)* positive zebrafish embryos were selected at 22 hpf. Immunoprecipitation was performed using Dynabeads protein G (Invitrogen) and anti-GFP monoclonal antibodies 19C8 and 19F7 (Heiman et al., 2008). Finally, RNA was purified using the Arcturus Picopure RNA isolation kit (Thermo Fisher) and the final yield was determined by Qubit 4 fluorometer. Three independent libraries were prepared from the RNA sample and sequenced with an Illumina HiSeq 2500 (2 × 125 cycles). At least 33 M reads were obtained for each library. Quality of reads was assessed by FastQC (v.0.72) and sequences corresponding to rRNA were removed using sortmerna-2.1. The remaining reads were aligned through Hisat2 (v.2.2.1) to the Danio rerio GRCz11 genome assembly. Counts were obtained using featureCounts (v.2.0.1). Finally, mRNA abundance was calculated in Reads Per Kilo base per Million mapped reads (RPKM) allowing the comparison among different samples. For the comparative expression analysis between whole-embryo (GEO accession number: GSM4521281) and *vsx2:TRAP* transcripts, we examined RPKM values for a list of transcription factor (TF) encoding genes that have been shown as enriched at the neural retina domain vs the RPE in cell populations isolated by FACS (Buono et al., 2021). To reduce noise, we included in our analysis only those genes with a RPKM value above 5 (89 TFs).

### Transgenic Lines Generation

The different transgenic lines were generated on the (AB/TU) background by microinjection of each vector (*Tol2_Vsx2.2:TRAP*, *Tol2_trap:TRAP* or *Tol2_UAS:TRAP*) into one-cell stage embryos. Following the Tol2 transposon/transposase transgenesis method (Kawakami et al., 2000), 100–200 pg of the plasmid were injected together with 100–200 pg of the *Tol2* transposase mRNA.

#### Trap-TRAP Lines

Embryos microinjected with the *Tol2_Trap:TRAP* construct were examined at 24 hpf and those showing any type of fluorescence were raised. When the selected fish reached adulthood, they were outcrossed with wild-type animals, and their progeny was examined at 24, 48, and 72 h post-fertilization (hpf) in order to identify and characterize the different *eGFP:rpl10a* expression patterns. Positive transgenic fish were considered as founders, and their progenies were raised to expand the different lines. In some cases, embryos from the same parental fish display different expression patterns, as a result of multiple integrations. In these cases, the different expression patterns were isolated through outcrossing and each different embryo was raised as an independent line. The integrity of the *eGFP:rpl10a* cassette at the landing loci was assessed by PCR using the specific primers TrapS_fwd: 5′-CAT​GTC​GAC​AAG​TGT​CCG, and TrapS_rev: 5′-TGC​ATT​CTA​GTT​GTG​GTT​TGT​CC).

#### UAS:TRAP Lines

Embryos microinjected with the *Tol2_UAS:TRAP* construct were also screened once they reached adulthood. To test the construct insertion and its functionality, microinjected fish were crossed with *Tg[Rx3:Gal4]* fish. To assess the expression pattern resulting from this cross, *Tg[Rx3:Gal4;UAS:TRAP]* embryos were compared with reference embryos from the *Tg[Rx3:Gal4;UAS:RFP]* line (Weiss et al., 2012).

### Imaging

The *eGFP:rpl10a* expression pattern displayed by the embryos of each trap-TRAP line was examined and photographed using an Olympus fluorescence micro stereoscope equipped with a camera Nikon. Pictures were taken at 24, 40, 48, and 72 hpf. To prevent embryo pigmentation and facilitate the analysis of their expression pattern, E3 medium was supplemented with 0.2 mM 1-phenyl-2-thiourea (PTU). In the case of the *UAS:TRAP* and the different Gal4 strains crosses, pictures of the progeny were taken at 24 h post fertilization. In all cases, the embryos were dechorionated with forceps if needed and mounted in 2% methylcellulose.

### Identification of Tol2_Trap:TRAP Integration Sites

From each analysed line (TT15, TT21, TT37, and TT42), genomic DNA was isolated from 5 individual 72 hpf zebrafish embryos using Chelex 100 sodium form (Sigma Aldrich). Briefly, embryos were incubated in 45 µl of Chelex 5% together with 5 µl of proteinase K (10 mg/ml) for a minimum of 2 h. Afterwards, proteinase K was heat-inactivated (95°C, 10 min). To prepare PCR templates, 5 µl (approximately 250 ng) of the extracted DNA was first digested using the restriction enzyme DpnII (NEB), diluted to 100 µl and ligated with T4 ligase (NEB). The resultant ligation was used as a template for inverse PCRs. Two different nested PCRs were performed to amplify the junction fragments containing both Tol2 ends and their adjacent genomic DNA. The first PCR product was diluted to 200 µl with ddH2O before the second PCR. The primers and PCR programs used were described in a previous study (Bessa et al., 2014). The common band amplified from the five embryos was purified and cloned in TOPO (Invitrogen, pCR8/GW/TOPO TA cloning KIT). The resulting clones were sequenced using the T7 primer and the sequences obtained were subsequently examined to identify the adjacent fragments, which were aligned to the zebrafish genome (GRCz10/danRer10) using the UCSC BLAT Search Genome tool (https://genome.ucsc.edu/cgi-bin/hgBlat) (Supplementary Table S1). The expression profile of nearby genes was then compared them with that of the corresponding line.

### Website

All information on the different trap:TRAP lines was uploaded to the zebrafish TRAP database website: trap-TRAP database: (https://amfermin.wixsite.com/website). Photographs of the different trap:TRAP lines at different developmental stages were included together with their descriptions and the trap:TRAP methodology.

## Results

### Vectors to Increase the Scope of the TRAP Technology in Zebrafish

**FIGURE 1:**
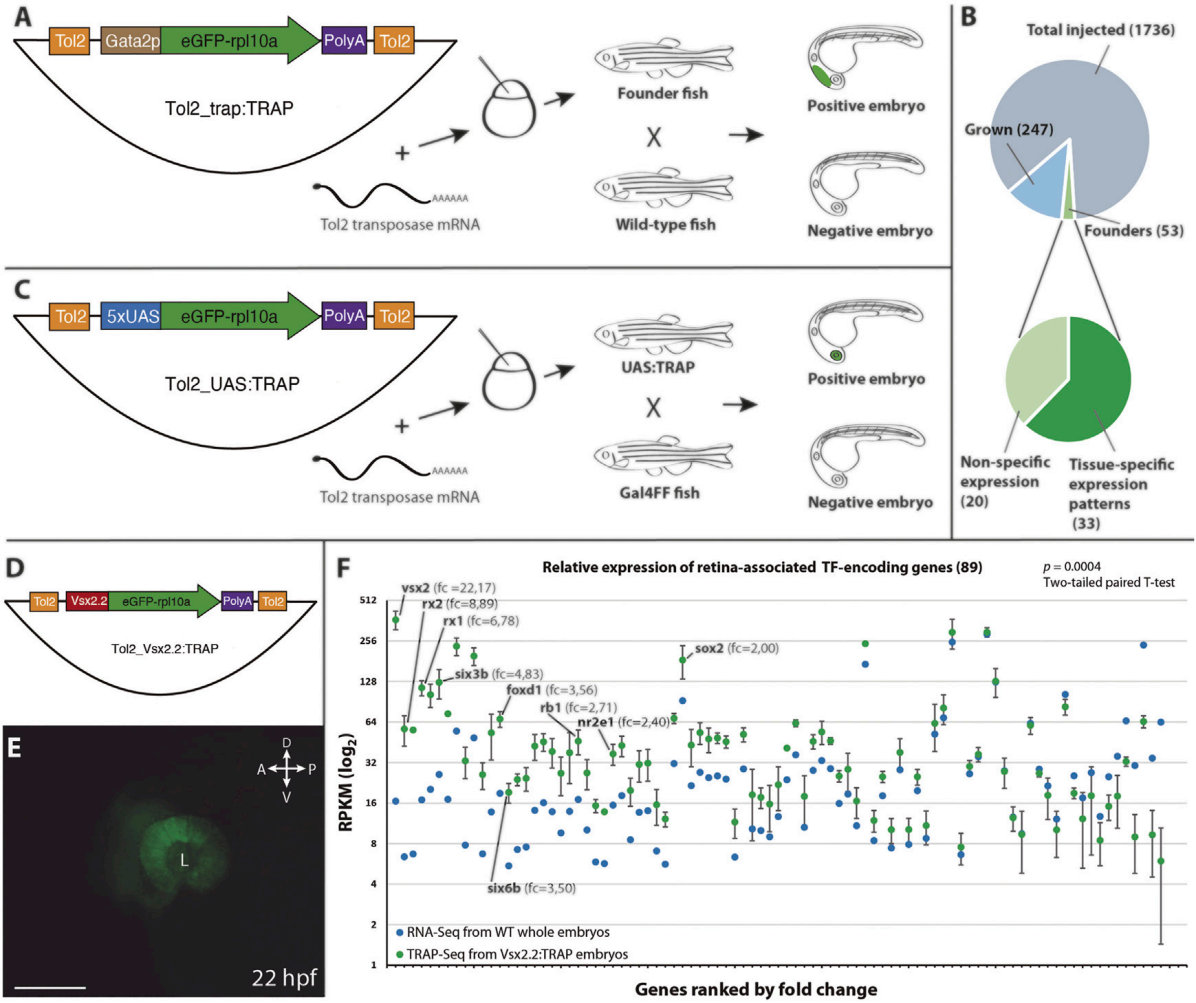
*Vectors and procedures used to expand TRAP approaches in zebrafish.* **(A)**: The *Tol2_trap:TRAP* vector comprises a cassette containing the *eGFP-rpl10a* fusion gene (green) and the gata2p minimal promoter (brown), flanked by Tol2 recognition sequences (orange). This vector was injected together with Tol2 transposase mRNA in one-cell stage zebrafish embryos. Once grown, adult fish were screened for eGFP-rpl10a expression in their progeny. **(B)**: Pie charts showing the efficiency rate of the trap:TRAP approach. **(C)**: The *Tol2_UAS:TRAP* vector comprises a cassette that contains the *eGFP-rpl10a* fusion gene (green) together with the 5xUAS element (blue), flanked by the Tol2 recognition sequences (orange). This vector together with *Tol2 transposase* mRNA were injected together in one-cell stage embryos. Once grown, adult fish were outcrossed with a Gal4 line to identify founders. **(D)**: Schematic representation of the *Tol2_vsx2.2:TRAP* vector used to test the functionality of the *eGFP-rpl10a* cassette (green) under the control of the *vsx2.2* promoter (red). **(E)**: Retina-specific eGFP-rpl10a expression in the line *Tg[vsx2.2:TRAP]* tested in TRAP-seq experiments at 22 hpf. L, lens. Scale bar = 100 µm. **(F)**: Differential expression (RPKM) of retinal TF-encoding genes in affinity-purified transcripts from *vsx2.2:TRAP* embryos at 22 hpf (TRAP-seq, *n* = 3, green), and in 22 hpf whole embryos RNA-seq sample (control, blue). Note the significant expression enrichment of neural retina-associated transcription factors (ranked by fold-change) in the *vsx2.2:TRAP* sample (*p = 0.0004*; two-tailed paired *t*-test). RPKM values corresponding to TFs from the core retinal GRN, as well as their fold change vs the control, are indicated.

To expand the range of potential applications of TRAP methods in zebrafish we designed two different Tol2-based vectors (Kawakami et al., 2000). The first, *Tol2_trap:TRAP* (Figure 1A), allows combining translating ribosome affinity purification (TRAP) with a standard enhancer trap approach. To this end, the TRAP cassette (*eGFP-rpl10a*) was fused to the *gata2p* minimal promoter (Bessa et al., 2009). The random integration of this vector in the zebrafish genome allowed capturing the activity of nearby cis-regulatory elements (i.e., enhancers), leading to the expression of *eGFP-rpl10a* in a tissue-specific manner. The second vector, *Tol2_UAS:TRAP* (Figure 1C), was designed to take advantage of the collection of Gal4 lines available in zebrafish (Abe et al., 2011) as drivers for the TRAP cassette. Both vectors were then tested in transgenic assays as described in the following sections.

To further examine the eGFP-rpl10a reported capacity to efficiently pull-down tissue-specific transcripts in zebrafish (Tryon et al., 2013), we fused this cassette to the neural-retina specific promoter *vsx2.2* (Nicolas-Perez et al., 2016; Buono et al., 2021). The resulting construct *Tol2_vsx2.2:TRAP* was microinjected into one-cell embryos to generate the corresponding zebrafish line *Tg[vsx2.2:TRAP]* (Figures 1D,E). Then, polysomes from 22 hpf *Tg[vsx2.2:TRAP]* embryos were harvested using a standard TRAP protocol, and captured RNA (120 ng) was deep sequenced (see Methods). To assess the retrieval efficiency of specific transcripts in that sample, we focused on the expression of a retina-enriched cluster of TFs (cluster 1), identified in our group in *vsx2.2*-positive retinal cells sorted by FACS at stage 23 hpf (Buono et al., 2021). We compared TFs relative mRNA abundance (RPKM) between affinity purified RNAs from *vsx2.2:TRAP* embryos at 22 hpf (TRAP-seq), and control RNA-seq samples obtained from whole embryos also at 22 hpf (GSM4521281). This analysis revealed a very significant enrichment (*p = 0.0004*; two-tailed paired *t*-test) of neural retina-specific transcription factors in the *vsx2.2:TRAP* sample (Figure 1F). Particularly high RPKM fold changes were observed for genes that belong to the core gene regulatory network of neural retina specification. This was the case for TF encoding-genes such as *vsx2* itself (22.2-fold), *rx2* (8.9-fold), *rx1* (6.8-fold), *six3b* (4.8-fold), *foxd1* (3.5-fold) or *six6b* (3.5-fold) (Figure 1F; Supplementary Table S2). These results confirm the functionality of the eGFP-rpl10a cassette for affinity purification of tissue-specific transcripts.

### Generation of the Trap-TRAP Transgenic Lines

To test TRAP compatibility with an enhancer trap approach we carried out a pilot screen in zebrafish. The *Tol2_trap:TRAP* vector was injected in one-cell stage zebrafish embryos together with Tol2 transposase mRNA synthesized *in vitro*. Embryos showing any fluorescence at 24 hpf were selected and raised. A total of 300 adult fish were screened for eGFP-rpl10a expression by outcrossing them with wild-type animals. A minimum of 100 F1 embryos from each cross was examined at 24 and 48 hpf. We identified 53 positive founders, indicating an enhancer trapping efficiency of 17.5% (Figure 1B). This efficiency rate falls within the range of previously reported trapping screens using the Tol2 transposition system: 12% for enhancer trapping (Parinov et al., 2004) and 23% for gene trapping (Kawakami et al., 2004). Among all founders, 33 (62% of total) showed tissue-specific expression patterns often restricted to a single domain. Each of these founders was isolated and outcrossed to generate stable lines, which were designated as TT followed by an identifier number. In most cases, the eGFP-rpl10a distribution indicated that the integration event was unique. However, some founders showed segregation of expression patterns in their progeny, indicating multiple integration events. This was the case for the lines TT1, which is derived from the same founder that produced the line TT56. Similarly, the 5 lines TT37, TT42, TT57, TT58, and TT59 derive from insertions found in the same founder; as it is also the case for the pairs TT48/TT50; and TT53/TT60 which are derived from common founders respectively. Among the stable lines, eGFP-rpl10a signal was detectable in a variety of tissues from different embryonic origin (i.e., from different germ layers) and located in different positions along the embryo axes. This observation indicates that the eGFP-rpl10a fusion does not compromise the random integration of the trapping cassette.

**FIGURE 2:**
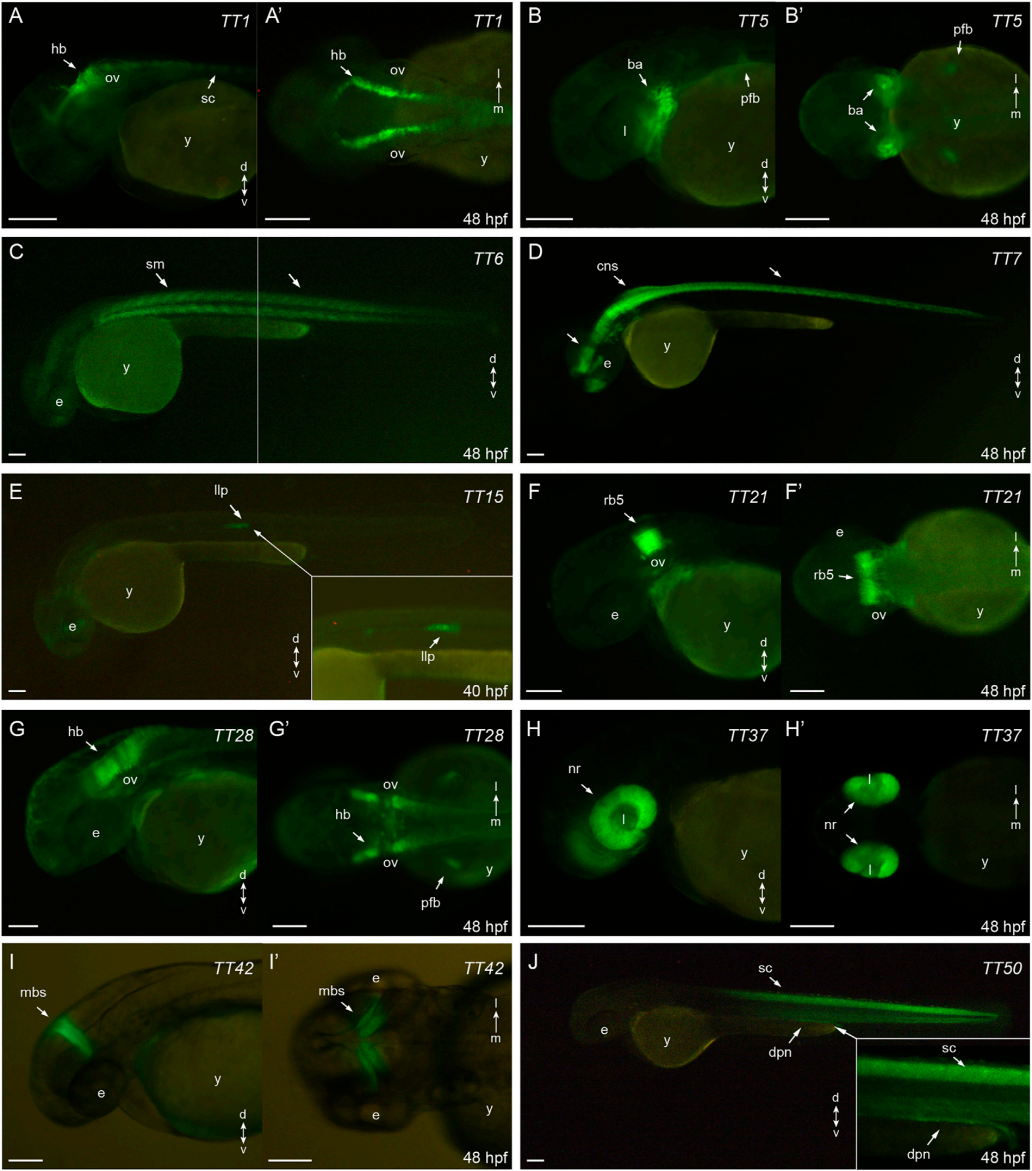
*eGFP-rpl10a expression patterns*: **(A,A′)**: TT1, hindbrain and spinal chord (lateral and dorsal, respectively); **(B,B′)**: TT5, jaw, brachial arches and pectoral fin buds (lateral and ventral, respectively); **(C)**: TT6, skeletal muscles (lateral); **(D)**: TT7, central nervous system (lateral); **(E)**: TT15, lateral line system (lateral); **(F, F′)**: TT21, rhombomere 5 (lateral and dorsal, respectively); **(G,G′)**: TT28, hindbrain and pectoral fin buds (lateral and dorsal, respectively); **(H,H′)**: TT37, retina (lateral and ventral, respectively); **(I,I′)**: TT42, midbrain stripe (lateral and dorsal, respectively); **(J)**: TT50, spinal cord and pronephros (lateral). ba, branchial arches; cns, central nervous system; dpn, distal pronephros; e, eye; hb, hindbrain; l, lens; llp, lateral line primordium; mbs, midbrain stripe; nr, neural retina; ov, otic vesicle; pfb, pectoral fin buds; rb5, rhombomere 5; sc, spinal cord, sm, skeletal muscles; y, yolk. Scale bar, 100 µm.

The expression patterns of the 10 most relevant transgenic lines (i.e. those exhibiting higher tissue specificity at 40–48 hpf) are shown in Figure 2. These include specific lines for hindbrain and spinal cord (TT1, Figures 2A, 2A′); jaw, branchial arches and pectoral fin buds (TT5, Figures 2B, 2B′); skeletal muscles (TT6, Figure 2C); central nervous system (TT7, Figure 2D); lateral line system (TT15, Figure 2E); rhombomere 5 (TT21, Figures 2F, 2F′); hindbrain and pectoral fin buds (TT28, Figures 2G, 2G′); retina (TT37, Figures 2H, 2H′); midbrain stripe (TT42, Figures 2I, 2I′) and spinal cord/pronephros (TT50, Figure 2J). For remaining lines, descriptions of their eGFP-rpl10a distribution patterns are shown in Supplementary Figures S1, S2. All the information on the trap-TRAP transgenic lines has been uploaded to a web-based database: trap-TRAP database. This website displays TT lines pictures and descriptions of their expression pattern at different developmental stages, together with complementary information about the trap-TRAP technology.

### Identification of Trap-TRAP Insertion Sites

**FIGURE 3:**
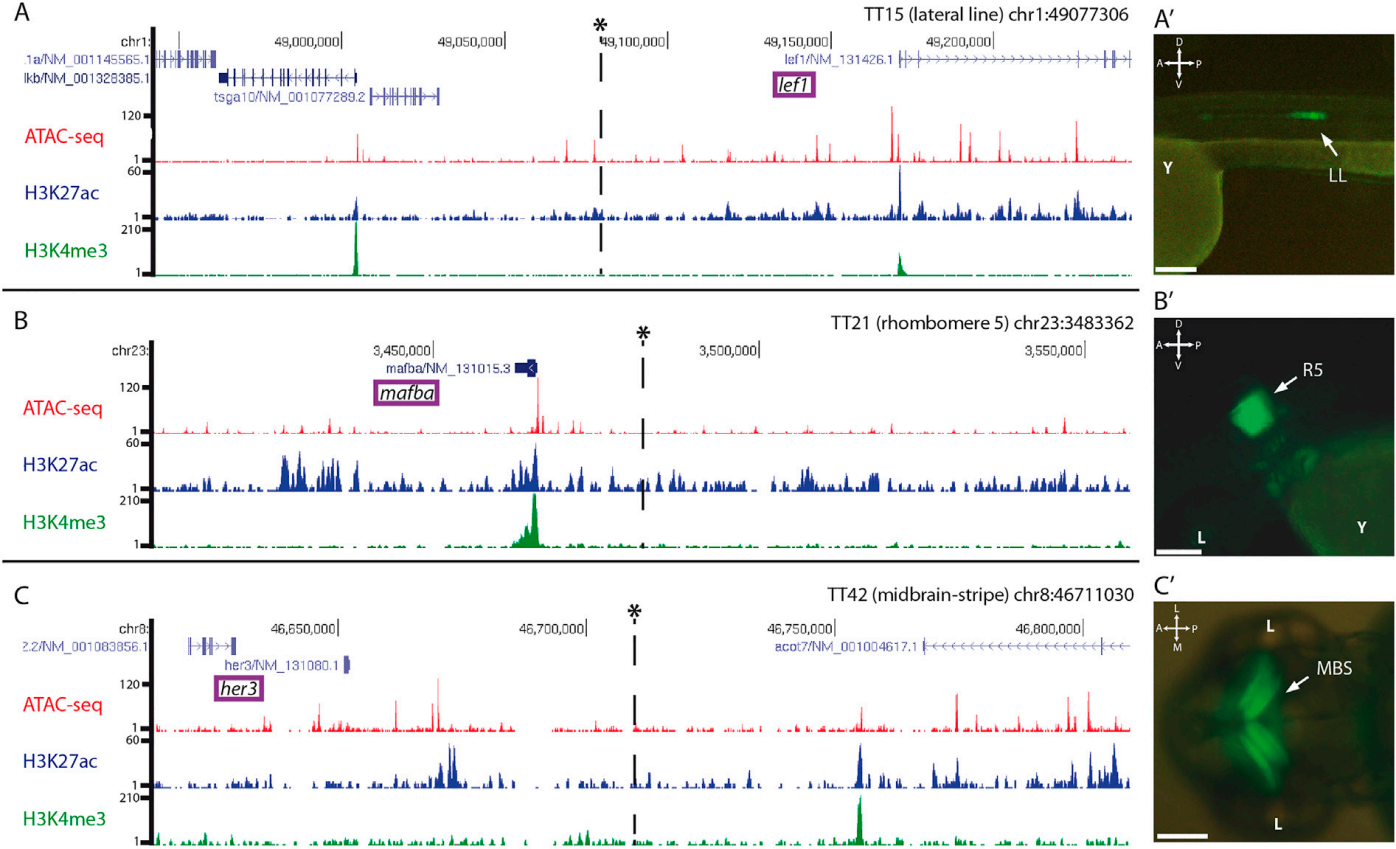
*Genomic insertion sites of trap:TRAP cassette in representative transgenic lines*: Insertions are marked with an asterisk*. Tracks for ATAC-seq, H3K27ac and H3K4me3 at 48 hpf are also shown for the different loci. **(A)**: Genomic location of the insert in the transgenic line TT15 in the vicinity of *lef1*, **(A′)**: TT15 expression pattern at 48 hpf. **(B)**: Genomic location of the insert in the transgenic line TT21 nearby *mafba*, **(B′)**: TT21 expression pattern at 48 hpf. **(C)**: Genomic location of the insert in the transgenic line TT42, **(C′)**: TT42 expression pattern at 48 hpf. LL, lateral line; R5, rhombomere 5; MBS, midbrain stripe. D-V, dorso-ventral; A-P, anterior-posterior. Scale bar = 100 µm.

To investigate if the random insertions of the trap-TRAP cassette in the genome recapitulate the activity of nearby cis-regulatory elements, we mapped the targeted loci of a few representative lines by inverse PCR and compared their expression patterns with those of neighbouring genes. The analysis provided conclusive mapping results for 3 out of 4 lines examined (Supplementary Table S1), the lines TT15, TT21, and TT42. In these cases, we successfully identified insertion sites in the vicinity of genes with an expression similar to that found for the corresponding strains (Figure 3). The line TT15, which showed expression in the lateral line, contains an insertion in the chromosome 1 (Figures 3A, 3A′). This region is 5′ distal to *lef1*, whose expression pattern includes a lateral line domain similar to that observed in TT15 embryos (Neelathi et al., 2018). Similarly, the analysis carried out on the TT21 line, which shows expression in rhombomere number 5, indicated that the insertion occurred on chromosome 23 (Figures 3B, 3B′). In the vicinity of the integration site is *mafba,* whose expression has been described in rhombomeres 5 and 6 (Moens et al., 1998). Finally, examination of the TT42 line identified an insertion on chromosome 8 (Figure 3C). This line shows a very specific expression stripe restricted to the midbrain (Figure 3C′). Accordingly, the neighbouring gene *her3* also shows expression in this domain (Thisse and Thisse, 2005). In all cases, the integration loci were compared with available data on chromatin accessibility, (ATAC-seq), as well as H3K27ac and H3K4me3 epigenetic marks (i.e., to highlight active enhancers and promoters respectively) previously obtained in 48 hpf zebrafish embryos (Bogdanovic et al., 2012; Marlétaz et al., 2018). These comparisons indicate that the eGFP-rpl10a cassette is able to capture the activity of nearby cis-regulatory elements.

### Compatibility of TRAP Technology With the Gal4/UAS System

**FIGURE 4:**
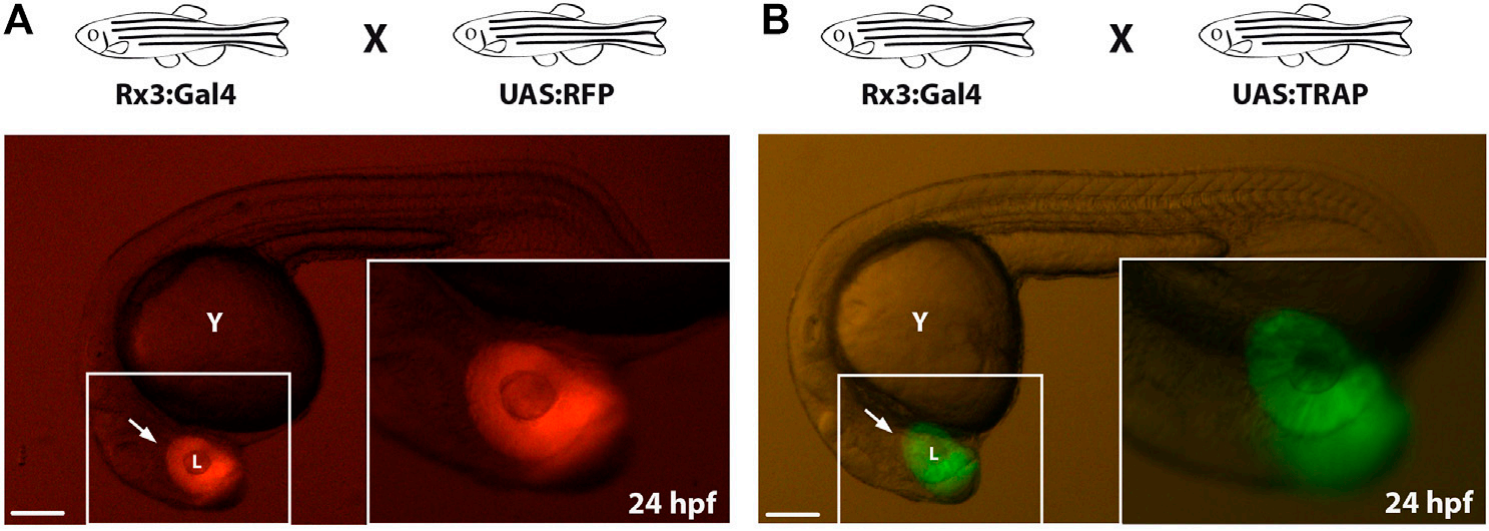
*Compatibility of TRAP technology with the Gal4/UAS system:* **(A)**: Zebrafish embryo from the control cross *Tg[Rx3:Gal4]* x *Tg[UAS:RFP]* showing RFP expression in the developing retina (arrow) at 24 hpf. **(B)**: zebrafish embryo derived from a *Tg[Rx3:Gal4]* × *Tg[UAS:TRAP]* cross showing *eGFP-rpl10a* expression at 24 hpf in the retina (arrow). l, lens; y, yolk. Scale bar = 100 µm.

As an alternative strategy to direct *eGFP-rpl10a* expression to tissues or cell types of interest, we combined the TRAP methodology with the Gal4-UAS system, which allows the spatiotemporal control of gene expression (Brand and Perrimon, 1993; Scheer and Campos-Ortega, 1999; Köster and Fraser, 2001). To this end, the fusion gene was placed under the control of the UAS element to generate the *Tol2_UAS:TRAP* vector (Figure 1C). This construct was injected in one-cell stage zebrafish embryos together with *in vitro -*synthesized Tol2 transposase mRNA. To screen for potential adult founders, F0 animals were outcrossed with fish from a Gal4-expressing strain: *Tg[Rx3:Gal4]*, whose expression is restricted to the retina as previously reported (Rembold et al., 2006; Weiss et al., 2012). As a reference control, we first verified retinal expression in the progeny of a *Tg[Rx3:Gal4]* X *Tg[UAS:RFP]* cross (Figure 4A). Then this retina-specific pattern was further confirmed in the founders progeny crossed to the Gal4 reference line: *Tg[Rx3:Gal4]* X *Tg[UAS:TRAP]* (Figure 4B). The fact that we did not observe toxicity, optic cup malformations, or developmental delays in these crosses indicates that Gal4 drivers can be successfully combined with the UAS:TRAP line to specifically perform TRAP analysis in a broad variety of tissues and cell types in zebrafish.

## Discussion

We have described two different approaches, Trap-TRAP and UAS-TRAP, which allow expanding the use of the TRAP methodology in zebrafish and can be easily adapted to other model organisms. While TRAP methods allow tissue-specific isolation of the mRNA fraction being translated, a closer representation of the protein profile of the cell at a given time (Heiman et al., 2014), the need to generate transgenic lines for each type of analysis has limited their application. Our strategies facilitate the fast and efficient generation of transgenic zebrafish strains suitable for TRAP analysis. Previous studies in zebrafish have reported the use of TRAP methods for translational profiling in zebrafish using a few tissue-specific promoters (Fang et al., 2013; Tryon et al., 2013; Housley et al., 2014; Houseright et al., 2020). Here we applied the Trap-TRAP strategy to generate random insertions of the *eGFP-rpl10a* cassette into the genome. In our pilot screen we examined 53 founders, 33 of them giving rise to tissue-specific stables lines, in whose progeny no developmental defects were observed, highlighting the compatibility of our approach with the large-scale generation of TRAP lines. Using a strategy similar to that of a previous study in *Drosophila* (Bertin et al., 2015), we also generated and tested a UAS:TRAP transgenic line that will allow to combine the TRAP methodology with the large collection of Gal4 lines currently existing in zebrafish. These two alternative approaches can facilitate the systematic generation of transgenic lines available for TRAP analysis. A potential limitation of them could be the sensitivity of the TRAP method when small cell populations are interrogated or weak promoters are employed or captured. However, the access to large numbers of synchronized embryos (i.e., once the stable lines have been generated), together with evidence from previous TRAP analyses in zebrafish targeting populations limited in number (Houseright et al., 2020), suggest that in many cases this should not be a major obstacle. A further refinement of the Trap-TRAP and UAS-Trap approaches may include the generation of transgenic lines harbouring an Avi-tagged *eGFP-rpl10a* cassette, as previously described (Housley et al., 2014). This system, in combination with BirA activating strains, does not depend on the use of specific antibodies for immunoprecipitation of tagged ribosomes and may result in an increased sensitivity.

## Data Availability

The datasets presented in this study can be found in online repositories. The names of the repository/repositories and accession number(s) can be found below: https://www.ncbi.nlm.nih.gov/geo/, GSE192511
