# Supplementary material for: Trap-TRAP, a Versatile Tool for Tissue-Specific Translatomics in Zebrafish
Source: Front Cell Dev Biol. 2022 Jan 31;9:817191. doi: 10.3389/fcell.2021.817191 (PMC8841413; doi:10.3389/fcell.2021.817191)
Supplement: Supplementary file 1 [file DataSheet1.PDF]

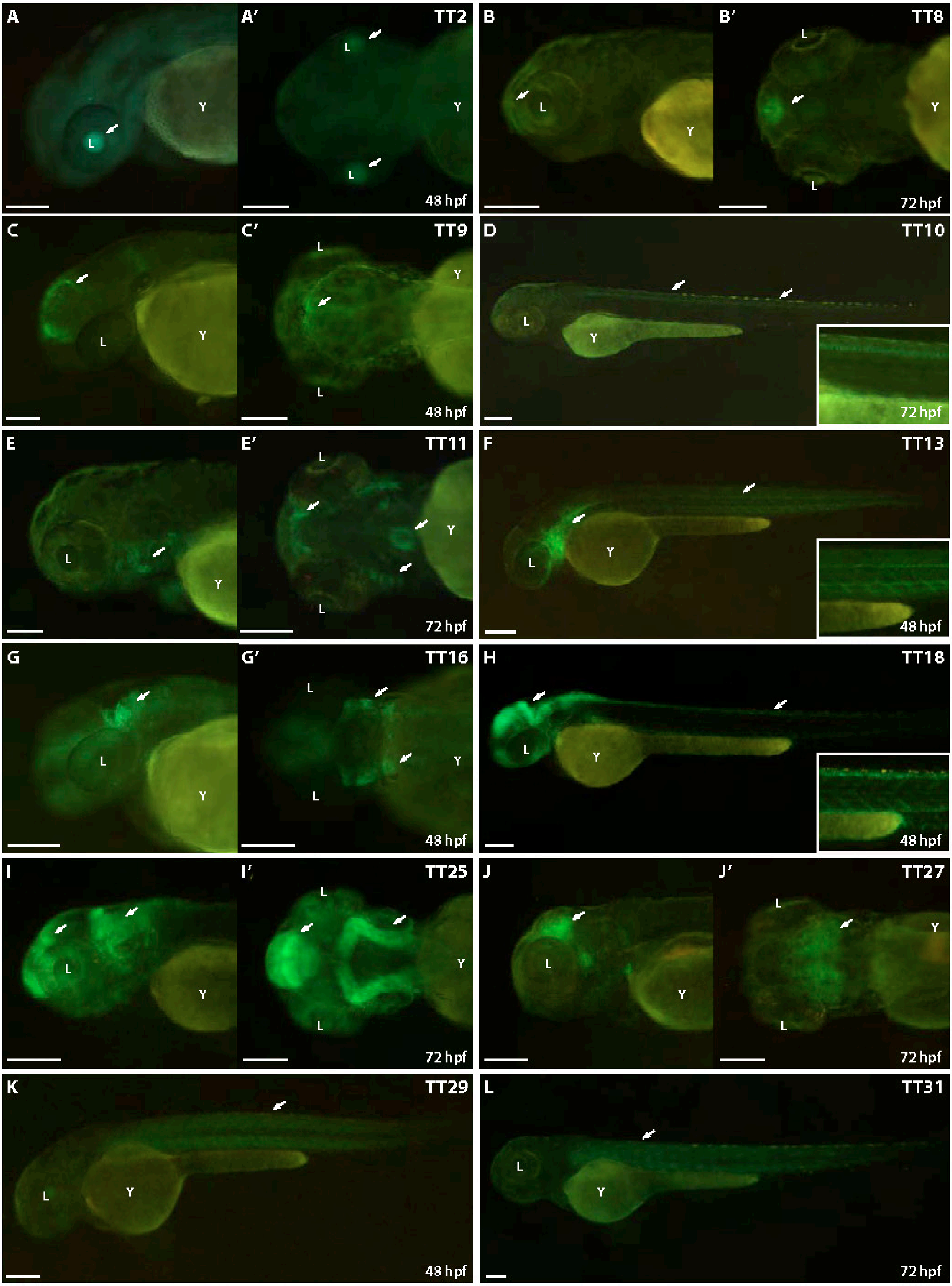

Figure Supplementary 1. eGFP-rpl10a expression patterns (TT2 - TT31). A, A′: TT2, lenses (lateral and dorsal, respectively); B, B′: TT8, forebrain, eyes (lateral and ventral, respectively); C, C′: TT9, dorsal optic tectum (lateral and dorsal, respectively); D: TT10, spinal chord neurons (lateral); E: TT11, branchial arches, jaw and heart (lateral and ventral respectively); F, F′: TT13, vascular system (lateral); G, G′: TT16, anterior hindbrain (lateral and dorsal, respectively); H, H′: TT18, Central nervous system, pectoral fin buds (lateral); I, I′: TT25, dorsal central nervous system, eye (lateral and dorsal, respectively); J, J′: TT27, midbrain (lateral and dorsal, respectively); K: TT29, lenses, skeletal muscles and central nervous system (dorsal); L: TT31 skeletal muscles (dorsal). l= lens; y = yolk. Scale bar = 100 µm.

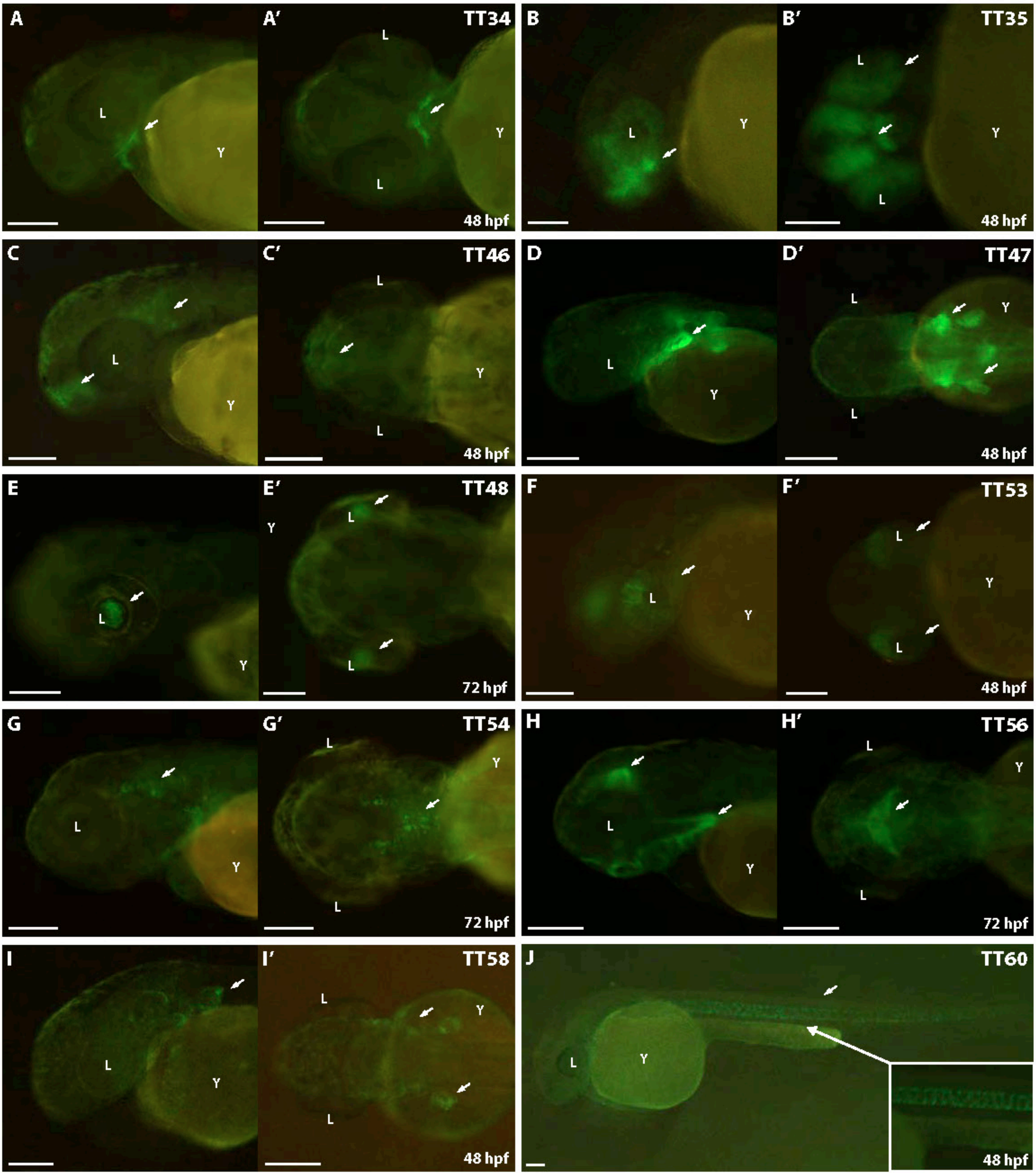

Figure Supplementary 2. eGFP-rpl10a expression patterns (TT34 - TT60). A, A': TT34, heart (lateral and dorsal, respectively); B, B': TT35, telencephalon, eyes (lateral and ventral, respectively); C, C': TT46, telencephalon (lateral and dorsal, respectively); D, D': TT47, pectoral fin buds, branchial arches (lateral and dorsal, respectively); E, E': TT48, lens fibers (lateral and ventral respectively); F, F': TT53, dorsal retina (lateral and ventral, respectively); G, G': TT54, ventral hindbrain (scattered cells), branquial arches (lateral and dorsal, respectively); H, H': TT56, midbrain subdomain, branchial arches, jaw (lateral and dorsal, respectively); I, I': TT58, pectoral fin buds, (lateral and dorsal, respectively); J: TT60, notochord (lateral); l= lens; y = yolk. Scale bar = 100 µm.

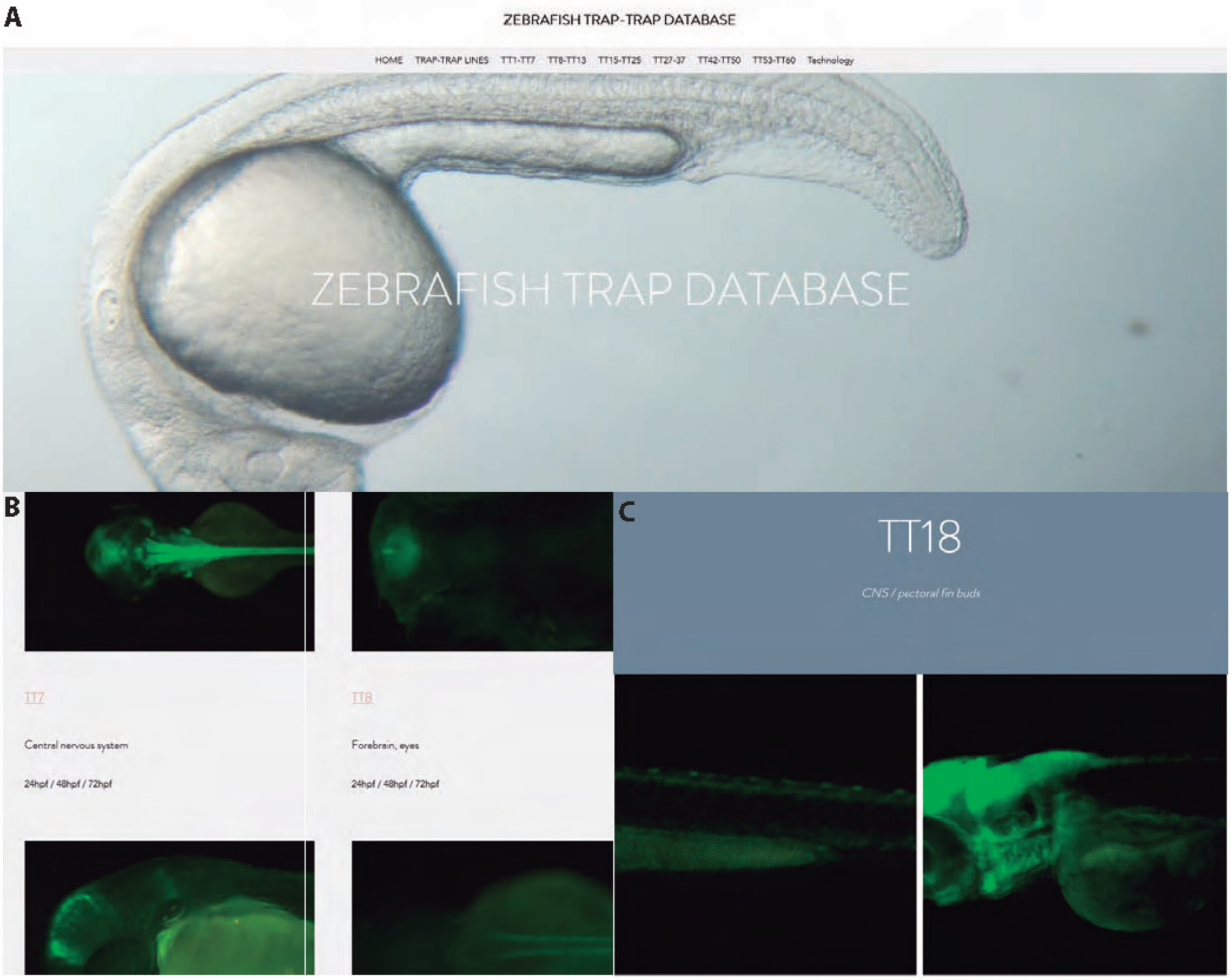

Figure Supplementary 3. Content of the zebrafish trap-TRAP database. (A) Homepage of the website. The menu at the top contains direct links to the different pages of the site. (B) Images of the summarizing page "trap-TRAP lines" with descriptions and links to each of the trap-TRAP lines. (C) Specific example for the tab displaying the TT18 line. Each tab shows a galary of images illustrating the expression pattern of an individual line.

Table S1. Analysed strains, corresponding cloned sequences matching genomic positions, and nearby 5'and 3' genes.

Strain	Sequence	5' gene	3' gene
**TT15**	tttaaatgtgcttttgttaaatcaaacttttg tttttttagaaaagattcataataaatgat	tsga10 https://zfin.org/ZDB-GENE-061013-482	lef1 https://zfin.org/ZDB-GENE-990714-26
**TT21**	ggaaatcagagcaggaaactctcctctgta attacccgaccaccagataaggcctacattag ctcgttctatctctctcagcatctgtcctctctttc tctcactttattcctgcgtgtgctttttaaggtgat aaagtgctcaacgcatacacaggaaaatggct taaaatgtttgataaagagaccagatgaaaata acattgaaga	mafba https://zfin.org/ZDB-GENE-980526-515	rnf114 https://zfin.org/ZDB-GENE-040813-1
**TT37**	Non conclusive	-	-
**TT42**	tcaccatgatattcggcaagcaggcatcgccatggg tcacgacgagatcctcgccgtcgggcatgctcgcctt gagcctggcgaacagttcggctggcgcgagcccctg atgttcttcgtccagatcatcctgatcgacaagaccg gcttccatccgagtacgtgctcgct	gpr153 https://zfin.org/ZDB-GENE-050823-7<br><br>her3 https://zfin.org/ZDB-GENE-980526-204	acot7 https://zfin.org/ZDB-GENE-040912-42

Table S2: RPKM values for neural retina-related TF-encoding genes, both in affinity-purified transcripts from vsx2.2:TRAP embryos at 22 hpf (TRAP-seq), and in 22 hpf whole embryos RNA-seq sample. TFs indicated in Fig 1F are marked in red.

GeneID	SYMBOL	Whole embryo RNA-seq_RPKM	TRAPseq_MEAN _RPKM	FoldChange (ranked)	TRAP1_RPKM	TRAP2_RPKM	TRAP3_RPKM
ENSDARG00000005574	vsx2	16,55956358	367,1664551	22,17247172	386,7200156	302,0558865	412,7234632
ENSDARG00000040321	rx2	6,393250501	56,81210561	8,886263037	40,3148873	67,04302571	63,07840383
ENSDARG00000019842	cnot3b	6,706504969	55,83533617	8,325549064	54,22908208	55,70071718	57,57620924
ENSDARG00000071684	rx1	16,92448123	114,8228391	6,784422961	112,2175704	101,4801062	130,7708406
ENSDARG00000006524	supt6h	20,22569066	102,0844243	5,047265186	109,486818	79,21214471	117,5543103
ENSDARG00000054879	six3b	26,07826066	126,0296923	4,832749161	113,2103106	103,1529469	161,7258195
ENSDARG00000045857	cebpz	17,08174392	73,65880358	4,31213604	74,40905796	72,13258748	74,4347653
ENSDARG00000079949	supt16h	54,773507	233,6939107	4,266550081	262,4149477	194,903536	243,7632485
ENSDARG00000034768	phf3	7,811105878	32,94699178	4,217967634	36,67243982	23,0959822	39,07255331
ENSDARG00000017397	smarcc1a	48,86636954	197,6332505	4,044361231	211,9441645	163,7028568	217,2527301
ENSDARG00000096428	si:dkey-217d	6,711268486	26,02705734	3,878112967	32,37758042	24,77440991	20,9291817
ENSDARG00000018907	kat6a	13,76218673	53,28836045	3,872085265	47,13370307	37,2270874	75,50429088
ENSDARG00000029179	foxd1	18,92574295	67,44311103	3,563564781	57,31317999	70,69827656	74,31787654
ENSDARG00000031316	six6b	5,481225995	19,21042612	3,50476812	22,88126683	16,9738143	17,77619724
ENSDARG00000008141	rbl1	7,239687789	23,95183792	3,308407575	23,37100302	26,59226065	21,8922501
ENSDARG00000086626	im:7147486	7,563200303	24,46610173	3,234887448	22,66959041	20,75672143	29,97199334
ENSDARG00000030665	tcerg1b	14,1510571	42,46784143	3,001036681	38,49296451	35,89759656	53,01296322
ENSDARG00000007092	xab2	16,10543479	45,6389627	2,833761603	48,79576624	50,36208079	37,75904108
ENSDARG00000009953	med14	13,83682042	38,62715007	2,791620394	40,29280873	28,38300706	47,20563442
ENSDARG00000016763	znf292a	9,607523334	26,63093958	2,771883934	28,44809432	17,38616273	34,05856168
ENSDARG00000062472	sin3b	13,8994432	37,89719503	2,726526127	44,06987648	20,25330537	49,36840325
ENSDARG00000006782	rb1	17,03153398	46,10270889	2,70690291	37,26672485	56,45207632	44,58932551
ENSDARG00000101060	gcna	10,10607642	26,84082405	2,655909469	23,20735252	34,62060696	22,69451268
ENSDARG00000101361	znf644a	5,844292195	15,30706793	2,619148294	17,22046021	13,90892223	14,79182135
ENSDARG00000012927	tcea2	5,695753107	13,79979317	2,422821515	14,24017184	13,58185471	13,57735296
ENSDARG00000017107	nr2e1	15,46929719	37,12055204	2,399627571	43,93784879	30,82785884	36,5959485
ENSDARG00000077013	znf280d	18,21707958	42,80641826	2,349795864	34,33436281	45,9212203	48,16367167
ENSDARG00000005867	gon4l	8,547849184	19,82117708	2,318849649	20,05554101	15,07084629	24,33714393
ENSDARG00000000567	znf281a	13,66913525	30,99072433	2,267204455	33,34270708	21,89978795	37,72967796
ENSDARG00000057940	dido1	14,05117634	31,64878615	2,252394062	37,8829648	22,18922431	34,87416934
ENSDARG00000098919	smarcc1b	7,069669347	15,57435434	2,202982003	15,80601445	10,82329882	20,09374975
ENSDARG00000079020	gcfc2	5,628801201	12,20155161	2,167699866	11,73565991	10,94438919	13,92460572
ENSDARG00000015921	pwp1	31,44533502	68,15899538	2,16753917	70,33471172	61,57392205	72,56835237
ENSDARG00000070913	sox2	92,30372077	184,6180942	2,000115409	194,1987399	129,1507072	230,5048356
ENSDARG00000090337	pprc1	21,5862774	43,12102006	1,997612616	54,39255947	28,46404331	46,50645741
ENSDARG00000035910	znf281b	27,09058384	53,3535033	1,969448263	49,7999039	45,85982451	64,40078149
ENSDARG00000019572	taf7	24,70488093	47,84015578	1,93646575	46,71712507	58,30969981	38,49364246
ENSDARG00000042835	tfdp2	25,45617317	48,69990557	1,913088242	52,08730106	43,7787174	50,23369824
ENSDARG00000026664	uri1	24,13040739	45,72391309	1,894867018	42,4595835	43,57539099	51,13676479
ENSDARG00000101569	ppargc1b	6,364828821	11,58433511	1,820054463	13,74487622	8,367738125	12,64039097
ENSDARG00000071694	ndc80	28,68627287	51,80726019	1,805994819	49,76868139	46,90280227	58,7502969
ENSDARG00000012499	per1b	10,30086523	18,45460922	1,791559137	12,42013906	12,90535619	30,03833241
ENSDARG00000026701	gtf2h1	10,01407442	17,65740182	1,763258498	15,84973752	15,912643	21,20982494
ENSDARG00000090998	si:cabz01071	9,010330609	15,73796948	1,746658382	13,51314197	11,23607165	22,46469483
ENSDARG00000077581	zzz3	12,70507234	21,9105775	1,724553541	14,3888088	21,24947651	30,09344718
ENSDARG00000037238	smad5	23,81563613	40,96759357	1,720197325	41,04306683	40,93664124	40,92307265
ENSDARG00000042796	yy1a	36,54386923	62,69640656	1,715647737	58,98749154	66,40586526	62,69586287
ENSDARG00000059812	sin3ab	10,58324512	17,96646267	1,69763267	22,94847431	9,267712377	21,68320132
ENSDARG00000015554	zic2a	28,01102341	45,88975858	1,638274971	49,75693241	39,44452193	48,46782139
ENSDARG00000078164	znf576.2	33,16384488	54,13649307	1,632394955	66,3561795	49,33262219	46,72067753
ENSDARG00000070463	e2f3	28,82923411	46,34497949	1,607568877	46,78957018	43,45652476	48,78884353
ENSDARG00000024598	homezb	15,92945168	25,41376122	1,595394602	27,01260668	22,51949173	26,70918525
ENSDARG00000008986	e2f7	18,65243067	28,49084956	1,527460419	36,49445845	18,78491098	30,19317926
ENSDARG00000079233	si:ch211-160	10,8818364	16,56572804	1,522328349	17,87668153	11,82150674	19,99899585
ENSDARG00000070019	taf15	171,652532	244,2894956	1,423162786	240,8974726	240,830976	251,1400382
ENSDARG00000045484	dcp1b	8,426651113	11,90590258	1,412886616	12,46735693	13,74070682	9,509643978
ENSDARG00000008119	si:ch211-195	18,14741473	25,0887563	1,382497544	27,7571039	23,07981436	24,42935063
ENSDARG00000055162	zhx2a	7,429546852	10,15127577	1,366338483	12,49484956	8,290238566	9,668739195
ENSDARG00000036855	plagx	28,23919189	38,01082162	1,346030785	26,86632902	40,64545721	46,52067864
ENSDARG00000079684	taf6l	7,884828944	10,17896865	1,29095618	12,67258091	8,758502064	9,10582299
ENSDARG00000014994	tbp	19,80585736	25,09361849	1,266979663	28,93120219	22,81081261	23,53884066
ENSDARG00000103871	si:ch73-109d	8,765805863	10,90682257	1,244246421	13,68638738	11,42196254	7,612117796
ENSDARG00000069910	gtf2f2a	52,00656552	62,60649051	1,203818977	64,34111306	85,61026563	37,86809284
ENSDARG00000042337	chrac1	68,79103	81,43460428	1,183796845	81,15325852	101,8074805	61,34307381

ENSDARG00000102452	eif3ha	250,7815333	296,4630569	1,182156648	227,1715938	373,420602	288,7969749
ENSDARG00000076657	plagl2	6,626279747	7,54705555	1,138958184	7,514258743	5,574238239	9,552669667
ENSDARG00000011000	gtf2a1	26,39039253	30,01280699	1,137262622	29,73249673	27,00759616	33,29832809
ENSDARG00000102590	polr3h	35,38597524	36,88551624	1,042376704	40,80803577	37,9235104	31,92500255
ENSDARG00000030479	hmgb1b	287,4036929	297,4728226	1,035034796	290,557876	323,3130928	278,547499
ENSDARG00000076509	polr2d	126,5964833	128,2990376	1,01344867	127,2633084	159,7104993	97,92330513
ENSDARG00000020043	cnot8	27,8070149	27,52721568	0,989937819	19,76233739	33,26246979	29,55683985
ENSDARG00000019566	neurod1	12,61000066	12,4502863	0,98733431	15,25732697	10,24028728	11,85324465
ENSDARG00000000568	ell	9,491817624	9,371125077	0,98728457	6,499867364	6,994173708	14,61933416
ENSDARG00000040926	nr2f2	62,54576093	60,45762007	0,96661419	63,75050859	50,59326325	67,02908836
ENSDARG00000044511	etv5b	28,58840245	26,64797307	0,932125295	27,49820517	27,60734186	24,83837218
ENSDARG00000071497	zic3	21,49492485	18,29391846	0,851080829	19,36231036	11,60795362	23,9114914
ENSDARG00000009594	nr1d2b	12,17233079	10,13668625	0,832764605	6,180506093	10,47961584	13,74993681
ENSDARG00000070475	khdrbs1b	102,6339454	83,14407898	0,810103116	73,9989906	80,05380504	95,37944129
ENSDARG00000069342	zgc:153115	25,53588977	18,97020215	0,74288393	17,26787193	20,73947805	18,90325647
ENSDARG00000070278	mettl14	17,50009548	12,22245364	0,698422112	10,7798267	6,09272881	19,7948054
ENSDARG00000101578	e2f4	26,9305628	18,16692403	0,674583898	7,407287093	30,43320577	16,66027922
ENSDARG00000087483	si:dkey-226l1	12,73637685	8,469339129	0,664972404	5,277527268	8,948532288	11,18195783
ENSDARG00000042004	nfya	25,1128966	15,14650048	0,603136338	18,85704332	13,03281897	13,54963916
ENSDARG00000019810	nfe2l3	35,61268243	18,04366635	0,506664062	26,79956191	14,55535846	12,77607867
ENSDARG00000074624	si:ch211-198	65,37202529	32,57098387	0,498240398	34,99034494	29,90782512	32,81478154
ENSDARG00000069289	gabpa	30,3930163	8,981548603	0,295513565	9,427218437	4,610975442	12,90645193
ENSDARG00000099175	hmgb1a	238,4664495	64,49171621	0,270443563	58,65594592	71,72346965	63,09573306
ENSDARG00000019293	tfdp1a	34,54463475	9,320346027	0,269805893	3,80036673	12,56554825	11,5951231
ENSDARG00000103316	si:ch211-212	63,9045848	5,948561903	0,093085057	11,08957789	2,644227848	4,111879971
